# Novel missense mutation in the *RSPO4* gene in congenital hyponychia and evidence for a polymorphic initiation codon (p.M1I)

**DOI:** 10.1186/1471-2350-13-120

**Published:** 2012-12-13

**Authors:** Tahir Naeem Khan, Joakim Klar, Sadia Nawaz, Muhammad Jameel, Muhammad Tariq, Naveed Altaf Malik, Shahid M Baig, Niklas Dahl

**Affiliations:** 1 Human Molecular Genetics Laboratory, Health Biotechnology Division, National Institute for Biotechnology and Genetic Engineering (NIBGE), Faisalabad, 38000, Pakistan; 2 Department of Immunology, Genetics and Pathology, The Rudbeck Laboratory and Science for Life Laboratory, Uppsala University, Uppsala, 751 85, Sweden

**Keywords:** Anonychia, Hyponychia, Mutation, *RSPO4* gene, Polymorphism

## Abstract

**Background:**

Anonychia/hyponychia congenita is a rare autosomal recessive developmental disorder characterized by the absence (anonychia) or hypoplasia (hyponuchia) of finger- and/or toenails frequently caused by mutations in the *R-spondin 4* (*RSPO4*) gene.

**Methods:**

Three hypo/anonychia consanguineous Pakistani families were ascertained and genotyped using microsatellite markers spanning the *RSPO4* locus on chromosome 20p13**.** Mutation screening of the *RSPO4* gene was carried out by direct sequencing of the entire coding region and all intron-exon boundaries.

**Results:**

Mutations in the *RSPO4* gene were identified in all families including a novel missense mutation c.178C>T (p.R60W) and two recurrent variants c.353G>A (p.C118Y) and c.3G>A (p.M1I). The c.3G>A variant was identified in unaffected family members and a control sample in a homozygous state.

**Conclusions:**

This study raises to 17 the number of known *RSPO4* mutations and further expands the molecular repertoire causing hypo/anonychia. The c.353G>A emerges as a recurrent change with a possible founder effect in the Pakistani population. Our findings suggest that c.3G>A is not sufficient to cause the disorder and could be considered a polymorphism.

## Background

The formation of nails is initiated in the upper limb followed by the development at the hindlimb and the morphogenesis is similar in primates and rodents [1]. This process is dependent on RSPO4 as shown by mutations in the *RSPO4* gene in autosomal recessive anonychia/hyponychia (MIM 206800) [2-7]. RSPO4 is a mediator of the highly conserved WNT signalling pathway and binds to the FZD family of receptors and the low-density lipo-protein related receptors (LRPs) 5 and 6 [8-10]. WNT signalling is critical for nail development and mutations in different WNT associated genes have been identified in disorders involving the nails. In addition to *RSPO4* mutations in anonychia/hyponychia, it was recently shown that mutations in *FZD6* cause isolated autosomal recessive nail dysplasia (MIM 614157) [11]. Furthermore, *WNT10A* (MIM 6062689) mutations are associated with odontoonychodermal dysplasia (OODD; MIM 257980) [12-14] and mutations in the WNT-associated transcription factors *LMX1B* (MIM 602575) and *MSX1* (MIM 142983) cause Nail-Patella (MIM 161200) and Witkop syndrome (MIM 189500), respectively [15,16].

We investigated three consanguineous families of Pakistani origin segregating autosomal recessive hyponychia of finger- and toenails. We report herein on three *RSPO4* missense mutations in a homozygous state of which one is novel. A second mutation is likely to be a founder mutation and a third variant, previously described as associated with anonychia, was identified in family members with normal nails as well as in a control individual.

## Methods

### Study subjects

**Figure 1:**
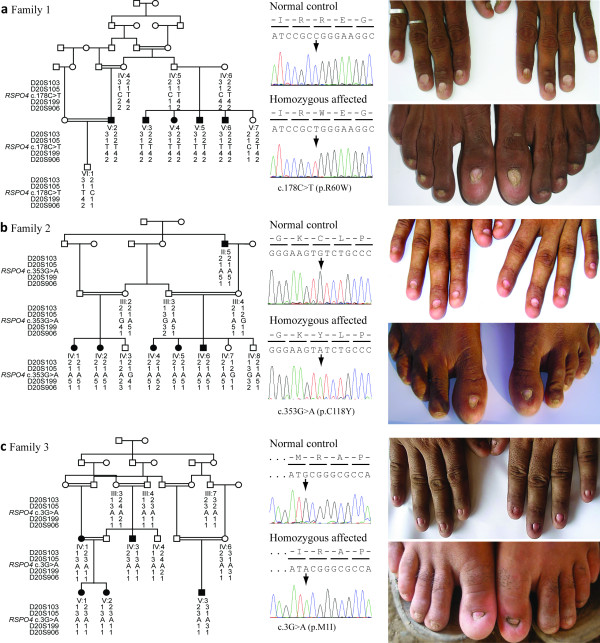
**Pedigrees, *RSPO4 *genotypes and phenotypes from three families segregating hyponychia.** Haplotypes of chromosome 20p13 markers are shown in the pedigrees below each symbol with the relative position of the *RSPO4* missense variants, respectively. (**a**) Family 1 comprises five affected individuals. Middle: Electropherogram showing the novel missense mutation c.178C>T (p.R60W). Right: Hands and feet of individual V:3. (**b**) Family 2 with six affected individuals. Middle: Electropherogram showing the missense mutation c.353G>A (p.C118Y). Right: Hands and feet of individual IV:4. (**c**) Family 3 comprises five affected individuals. Middle: Electropherogram showing the missense variant c.3G>A (p.M1I). Right: Hands and feet of individual IV:3.

Three consanguineous pedigrees of Pakistani origin with several members affected with hyponychia were investigated (Figure 1a-c). The nail phenotype ranged from almost complete absence of some nail plates to reduced size of nail plates. Affected individuals had no other ectodermal abnormality including the teeth, skin and hair and they reported normal sweating. Informed consent was obtained from all individuals who participated in this study under a protocol approved by the local ethical committee at National Institute for Biotechnology and Genetic Engineering (NIBGE), Faisalabad, Pakistan.

### Genotyping

Blood samples from a total of 32 available members from the three families were obtained and DNA was extracted using standard protocols. All family members were initially genotyped using microsatellite markers D20S103, D20S105, D20S199 and D20S906 spanning the *RSPO4* gene on chromosome 20p13**.** Mutation screening of the *RSPO4* gene included all five exons and intron-exon boundaries using Big Dye Terminator v3.1 cycle sequencing kit (Applied Biosystems, Foster City, CA) and separated on an ABI 3730xl DNA analyzer (Applied Biosystems). Segregation of each mutation was confirmed by screening all available family members. Effects of missense variants were predicted using PolyPhen-2 ( http://genetics.bwh.harvard.edu/pph2/).

## Results

Haplotype analysis and autozygosity mapping using microsatellite markers flanking *RSPO4* confirmed linkage to 20p13 in families 1 and 2 (Figure 1a-b). In family 3, the results suggested linkage (Figure 1c). Sequence analysis of the *RSPO4* gene in affected members of family 1 revealed a novel missense variant c.178C>T. The mutation was found in a homozygous state in all 5 affected individuals and in a heterozygous state in their parents (Figure 1a). The mutation is located in exon 2 and results in a p.R60W substitution. The replacement for the neutral non-polar tryptophan is predicted to be damaging with a PolyPhen-2 score of 0.999. The mutation was excluded on 192 control chromosomes from 96 healthy Pakistani individuals. In family 2 we identified a missense variant c.353G>A resulting in a p.C118Y substitution in a homozygous state in all affected individuals (Figure 1b). The mutation is predicted to be damaging with a PolyPhen-2 score of 0.96. In family 3 we found the sequence variant c.3G>A resulting in p.M1I in five affected individuals as well as in five family members with normal finger- and toenails (Figure 1c). The amino-acid substitution is predicted as possibly pathogenic with a PolyPhen-2 score of 0.88 and both methionine and isoleucine belong to the group of neutral and non-polar amino acids. We screened 96 healthy Pakistani controls for this sequence variant and we identified one homozygous individual. This corresponds to an allele frequency of 1.1% for p.M1I. Non-coding variants in the *RSPO4* gene were excluded by sequencing the entire 5’ and 3’ UTRs as well as a predicted promoter region one kb upstream of the start codon in affected individuals.

## Discussion

The majority of cases affected by isolated or non-syndromic congenital anonychia/hyponychia reported so far are caused by mutations in the *R-spondin 4* (*RSPO4*) gene [2-7]. *RSPO4* is expressed in mesenchymal cells from which the nails are derived [2] and the RSPO4 protein act as a Frizzled (FZD) agonist in WNT signalling. The importance of WNT signalling for nail development was further supported by the recent identification of *FZD6* mutations in autosomal recessive nail dysplasia [11].

**Figure 2:**
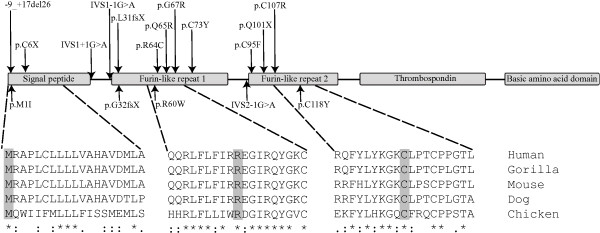
**Schematic illustration of the R-spondin 4 (RSPO4) structure with functional domains (boxed) and the relative positions of the 17 mutations known to date in anonychia/hyponychia [2–7 and this study].** The predicted effects of 13 coding mutations on RSPO4 are indicated. Four additional variants involve non-coding regions, i.e. one deletion and three splice site mutations, and the genomic positions for these variants are given arbitrary. The five protein domains are encoded by five corresponding exons in the *RSPO4* gene. Three missense variants identified in this study (p.M1I, p.R60W and p.C118Y) are all positioned in functional domains of which p.M1I should be considered a polymorphism. The degree of conservation across various species is shown for regions around the residues p.M1, p.R60 and p.C118 indicated by shaded areas, respectively (bottom). Notably, the signal peptide domain is less conserved than the furin-like repeats.

In this study, we analysed three consanguineous pedigrees segregating hyponychia. In each family the affected members were found homozygous for a distinct *RSPO4* missense variant. In one family we found a novel mutation that results in a p.R60W substitution in the first furin-like domain associated with the disease. The finding is consistent with the suggestion that both furin-like domains are required for inducing β-catenin stabilization [5]. Furthermore, the positively charged arginine residue is highly conserved among different species (Figure 2). In a second family we identified a p.C118Y substition in a homozygous state in affected individuals. This mutation has been reported previously in an independent Pakistani pedigree with hyponychia [2] suggesting a founder effect for the p.C118Y mutation in the Pakistani population. In a third family we identified a p.M1I variant in five affected individuals as well as in five members with a normal nail phenotype. Moreover, we also found the mutation in a homozygous state in one Pakistani control individual (1.1% allele frequency). Thus, the p.M1I variant, previously observed in two Pakistani siblings with anonychia [5], is not sufficient to cause the disorder and may represent a rare polymorphism. We hypothesize that the RSPO4 protein uses the second methionine codon at position 17 in the first exon for translational initiation. This will result in a shorter protein missing part of the signal peptide of importance for the secretory pathway [17]. However, the signal peptide is less conserved across species than the furin-like domains (Figure 2) and may not be required for normal nail development. This is supported by the fact that the furin-like domains of RSPO4, encoded after codon 17, are sufficient for inducing β-catenin stabilization [5].

With this report, the total number of *RSPO4* mutations associated with anonychia/hyponychia is expanded to 17, all of which are located in the first three exons encoding a signal peptide and the highly conserved furin-like, cystein-rich domains. Furthermore, we have identified a recurrent mutation in the Pakistani population and we suggest that the p.M1I variant could be considered a polymorphism as it is not sufficient to cause anonychia/hyponychia.

### Consent

Patients and their guardians provide written consent for publishing photographs and other material.

## Conclusions

We have identified one novel and one recurrent missense mutation in the *RSO4* gene associated with congenital hyponychia. Furthermore, our findings indicate that the RSPO4 missense variant p.M1I is a polymorphism despite its previous association with anonychia in two Pakistani siblings. We postulate that the common translational start for RSPO4 may be replaced by the second methionine codon (p.M17). The combined results improve our current knowledge about *RSPO4* in nail development of both diagnostic and biological importance.

## Abbreviations

RSPO4: R-spondin 4; NIBGE: National Institute for Biotechnology and Genetic Engineering; MIM: Mendelian inheritance in man; FZD: Frizzled; WNT10A: Wingless-type MMTV integration site family, member 10A; LMX1B: LIM homeobox transcription factor 1-beta; MSX1: Msh homeobox 1.

## Competing interests

The authors declare that they have no competing interests.

## Authors’ contributions

TNK and JK carried out molecular genetic studies including autozygosity mapping and sequencing for two families and the controls and drafted the manuscript. SN, MJ, MT and NAM investigated the families, collected samples and extracted DNA. SMB and ND designed and supervised the study, analysed the data and finalized the manuscript. All authors read and approved the final manuscript.

## Pre-publication history

The pre-publication history for this paper can be accessed here:

http://www.biomedcentral.com/1471-2350/13/120/prepub
